# Cytoskeleton Reorganization as an Alternative Mechanism of Store-Operated Calcium Entry Control in Neuroendocrine-Differentiated Cells

**DOI:** 10.1371/journal.pone.0045615

**Published:** 2012-09-25

**Authors:** Karine Vanoverberghe, V’yacheslav Lehen’kyi, Stéphanie Thébault, Maylis Raphaël, Fabien Vanden Abeele, Christian Slomianny, Pascal Mariot, Natalia Prevarskaya

**Affiliations:** 1 Inserm, U-1003, Equipe labellisée par la Ligue Nationale contre le cancer, Villeneuve d’Ascq, France; 2 Laboratory of Excellence, Ion Channels Science and Therapeutics, Université des Sciences et Technologies de Lille (USTL), Villeneuve d’Ascq, France; Ospedale Pediatrico Bambino Gesu’, Italy

## Abstract

Neuroendocrine differentiation (NED) is a hallmark of advanced androgen-independent prostate cancer, for which no successful therapy exists. NED tumour cells escape apoptotic cell death by alterations of Ca^2+^ homeostasis where the store-operated Ca^2+^ entry (SOCE) is known to be a key event. We have previously shown that the downregulation of Orai1 protein representing the major molecular component of endogenous SOCE in human prostate cancer cells, and constituting the principal source of Ca^2+^ influx used by the cell to trigger apoptosis, contributes to the establishment of an apoptosis-resistant phenotype (Cell Death Dis. 2010 Sep 16;1:e75.). Here, we report for the first time that the decrease of SOCE during NED may be caused by alternative NED-induced mechanism involving cytoskeleton reorganisation. NED induced by androgen deprivation resulted in a decrease of SOCE due to cortical F-actin over-polymerization which inhibits thapsigargin-induced SOCE. The disruption of F-actin polymerization by Cytochalasin D in NED cells restored SOCE, while the induction of F-actin polymerization by jasplakinolide or calyculin A diminished SOCE without changing the expression of key SOCE players: Orai1, STIM1, and TRPC1. Our data suggest that targeting cytoskeleton-induced pathways of malignant cells together with SOCE-involved channels may prove a useful strategy in the treatment of advanced prostate cancer.

## Introduction

Neuroendocrine differentiation (NED) is a process which occurs in several types of carcinomas. Prostatic carcinoma belongs to the tumours in which NED is suggested as an indicator of poor prognostic as the rate of NED of prostate cancer cells increases with the grade of the prostate cancer [1], [2], [3]. Even though NED is the subject of intense research, the molecular and cellular mechanisms involved in this process remain unclear.

Prostate cancer (PCa), the second cause of cancer-related men disease in western countries, depends on androgen in the early stages. Androgen deprivation therapies also cause prostate tumour regression. However, such treatments become unsuccessful when PCa cells progress to an androgen-independent stage [4]. Therefore, assessing the mechanisms occurring during the evolution to androgen-independence appears to be crucial. We and others have previously demonstrated that the androgen-dependent LNCaP (Lymph Node Carcinoma of the Prostate, [5] PCa epithelial cells acquire NED characteristics in response to long-term androgen ablation [6], [7], [8].

It is well known that as NED cells are androgen-independent [9] and characterized by an apoptotic cell death resistance [10], [11]. Tumour enrichment in NED cells enhances the malignant potential and drastically affects cancer cell responsiveness to androgen ablation therapy [12]. Normally present in the healthy prostate where they participate in the development and in the regulation of secretary processes of the mature gland, NED cells display structural, functional and morphological characteristics of neurons [12], [13], [14]. Their resistance to apoptosis does not seem to involve anti-apoptotic oncoprotein bcl-2 overexpression but rather the discovered survival proteins such as survivin and clusterin [15], [16], [17]. Despite these data, continuing effort is required to determine all characteristic features of NED cell apoptosis-resistance in an attempt to find new targets for therapeutic intervention in advanced PCa.

We previously demonstrated that NED of LNCaP cells induced alterations in Ca^2+^ homeostasis including reduced filling of the endoplasmic reticulum (ER) Ca^2+^ store, decreased expression of the endolemmal SERCA 2b Ca^2+^ ATPase and the luminal Ca^2+^ binding calreticulin and down-regulated store-operated current (I_SOC_) [18]. Moreover, NED LNCaP cells display a thapsigargin- (Tg) induced apoptosis resistance. It is well established that Tg induces apoptosis by depleting ER Ca^2+^ store followed by a store-operated or capacitative Ca2+ entry (SOCE or CCE) carried by SOC channels [19]. One of them Orai1, represents the major molecular component of endogenous SOCE in human PCa cells, and constitutes the principal source of Ca^2+^ influx used by the cell to trigger apoptosis [20]. The downregulation of Orai1, and consequently SOCE, protected the cells from diverse apoptosis-inducing pathways, such as those induced by thapsigargin (Tg), tumour necrosis factor α, and cisplatin/oxaliplatin. Orai1 rescue, following Orai1 transfection of steroid-deprived cells, re-established the store-operated channel current and restored the normal rate of apoptosis, suggesting its role in the establishment of an apoptosis-resistant phenotype in PCa cells. Thus, the apoptosis resistance of androgen-independent PCa cells is associated with the downregulation of Orai1 expression as well as SOCE. On the other hand, the TRP (transient receptor potential) proteins family which is widely distributed in mammalian tissues [21] is currently known to play an crucial role in the generation of SOCE. Even though their physiological mechanisms of activation and regulation are still unclear, several processes have been proposed to explain SOCE regulation: at the level of TRP proteins expression, or TRP channel activity regulation by accessory proteins [22], [23], or by TRPC channels internalization/membrane insertion caused by cytoskeleton reorganization [24], [25], [26], [27], [28], [29]. Indeed, it has been reported that TRPC channels activity involves their integration in plasmalemmal signaling complexes [30], [31], whose stability depends at least on cytoskeleton integrity, especially the actin polymerization. In this respect and as TRP channels and Orai1 play a key role in SOCE which is one of the pro-apoptotic pathway in NED cells we sought to study how cytoskeleton rearrangements may influence the activity of SOCE, the NED itself, and consequently apoptosis resistance of PCa cells.

## Materials and Methods

### Cell Culture

LNCaP cells from the American Type Culture Collection were cultured in RPMI 1640 medium (Biowhittaker, Fontenay sous Bois, France) supplemented with 5 mM L-glutamine (Sigma, L’Isle d’Abeau, France) and 10% fetal bovine serum (Seromed, Poly-Labo, Strasbourg, France). The culture medium also contained 50,000 IU/l penicillin and 50 mg/l streptomycin. Cells were routinely grown in 50 ml flasks (Nunc, Poly- labo) and kept at 37°C in a humidified incubator in an air/CO2 (95/5%) atmosphere. For Ca2+ imaging experiments, the cells were subcultured in Petri dishes (Nunc) coated with polyornithine (Sigma, 5 mg/l) and used after 3 to 6 days. Prior to fluorescence measurements, the cells were removed from the culture flasks with 0.05% trypsin (Life Technologies, Cergy Pontoise, France) and cultivated on glass coverslips in the same culture medium.

### Charcoal Stripped Medium

Tubes containing charcoal 10% (w/v) and FBS were agitated for 16 hours at 4°C. Following 1 hour of centrifugation at 10000 g and 4°C, the supernatant was collected and centrifuged again for 30 min at 27000 g. The resultant supernatant was filtrated through 0.22 µm filters. Before use, the charcoal stripped FBS was decomplemented for 30 min at 56°C. The charcoal stripped culture was obtained using charcoal stripped FBS and RPMI 1640 without Phenol Red, for 4 days.

### Calcium Imaging

[Ca^2+^]_i_ was measured using fura-2 (the detailed procedure has been described previously, [32]). The extracellular solution contained: NaCl-120, KCl-6, CaCl_2_-2, MgCl_2_-2, HEPES-10, and glucose-12. For Ca^2+^-free HBSS, CaCl_2_ was removed and EGTA (0.5 mM) added.

### Electrophysiological Recordings

Membrane currents in LNCaP cells were recorded in the whole-cell configuration using the patch-clamp technique, and also using a computer-controlled EPC-9 amplifier (HEKA Electronic, Germany) as described previously [33] Patch pipettes were made on a P-97 puller (Sutter, USA) from borosilicate glass capillaries (WPI, USA). Extracellular solution used to record Ca^2+^-carried I_SOC_ contained (in mM): 120 NaCl, 5 KCl, 10 CaCl_2_, 2 MgCl_2_, 5 glucose, 10 HEPES (pH adjusted to 7.3 with TEA OH). The pipettes were filled with the basic intracellular pipette solution (in mM): 120 Cs Methane sulfonate, 10 CsCl, 10 HEPES, 10 BAPTA, 6 MgCl_2_ (pH adjusted to 7.2 with CsOH).

### Immunostaining

Cells were cultivated on 14 mm diameter glass coverslips. After 4 days of culture, cells were rinsed once with PBS (pH7.5), fixed with 4% formaldehyde-1X PBS for 30 min, treated with 100 mM glycine-PBS for 30 min, permeabilized with methanol triton 1% for 20 min, and then washed three times with PBS. After washing, the samples were blocked with donkey serum for 30 min, then incubated with the FITC-phalloidin (1∶100) 1 hour at 37°C or overnight when cells are treated with pharmacological compounds. After washing, samples were then mounted on glass slides. Slides were examined on a confocal microscope (Zeiss LSM 510) equipped with an argon-krypton laser; 488 nm light was used for the excitation of the FITC-phalloidin, and images were collected using a 40X oil immersion objective.

### Western-blotting

Subconfluent LNCaP cells were treated with an ice-cold lysis buffer containing: 10 mM Tris-HCl, pH 7.4, 150 mM NaCl, 10 mM MgCl, 1 mM PMSF, 1% Nonidet P-40, and protease inhibitor cocktail from Sigma. The lysates were centrifuged 15,000×g at 4°C for 20 minutes, mixed with a sample buffer containing: 125 mM Tris-HCl pH 6.8, 4% SDS, 5% β-mercaptoethanol, 20% glycerol, 0.01% bromphenol blue, and boiled for 5 min at 95°C. Total protein samples were subjected to 10% SDS-PAGE and transferred to a nitrocellulose membrane by semi-dry Western blotting (Bio-Rad Laboratories). The membrane was blocked in a 5% milk containing TNT buffer (Tris-HCl, pH 7.5, 140 mM NaCl, and 0.05% Tween 20) overnight then probed using specific rabbit polyclonal anti-hStim1 (1∶250, BD Transduction Laboratories) and mouse monoclonal anti-β-actin (Lab Vision Co.) antibodies. The bands on the membrane were visualized using enhanced chemiluminescence method (1∶1000, Pierce Biotechnologies Inc.). Densitometric analysis was performed using a Bio-Rad image acquisition system (Bio-Rad Laboratories).

### RT-PCR

Total RNA was isolated using the guanidium thiocyanate-phenol-chloroform extraction procedure. After DNase I (Life Technologies) treatment to eliminate genomic DNA, 2 µg of total RNA was reverse transcribed into cDNA at 42°C using random hexamer primers (Perkin Elmer) and MuLV reverse transcriptase (Perkin Elmer) in a 20 µl final volume, followed by PCR as described below. The PCR primers used to amplify hStim1, hOrai1, Orai2, Orai3 cDNAs as well as the primers for β-actin are specified in Table 1. PCR was performed on the RT-generated cDNA using a GeneAmp PCR System 2400 thermal cycler (Perkin Elmer). To detect different cDNAs, PCR was performed by adding 1 µl of the RT template to a mixture of (final concentrations): 50 mM KCl, 10 mM Tris-HCl (pH 8.3), 2.5 mM MgCl2, 200 µM of each dNTP, 600 nM of sense and antisense primers, and 1 U AmpliTaq Gold (Perkin Elmer) in a final volume of 25 µl. DNA amplification conditions included an initial 5 min denaturation step at 95°C (which also activated the Gold variant of Taq Polymrase), and 33 cycles of 30 sec at 95°C, 30 sec at 59°C, 30 sec at 72°C, and finally 7 min at 72°C. Then, density measurements were performed with “Quantity one” software (Biorad) and the data were analyzed using Origin 7.0 (Microcal Software Inc., Northampton, MA, USA).

**Table 1:** Primers used for the for semiquantitative PCR and the real-time quantitative PCR (*in italic script*).

No		Name(Accession No)		Forward(5′-…- 3′)	Backward(5′-…- 3′)	Expected Size (b.p)	
1.		hSTIM1 (NM_003156)		GCGGGAGGGTACTGAG	TCCATGTCATCCACGTCGTCA	533	
2.		hOrai1 (NM_032790)		CTTCAGTGCCTGCACCACAG	CCTGGAACTGTCGGTCAGTC	450	
3.		hOrai2 (NM_032790)		GGAGACGCAGTACCAGTACC	GTGAAGACCACGAAGATGAGG	395	
4.		hOrai3 (NM_032790)		CTGGAGAGTGACCACGAGTAC	GAGATTGGAAGCTGGACTAAG	380	
5.		β-Actin (NM_001101)		CAGAGCAAGAGAGGCATCCT	GTTGAAGGTCTCAAACATGATC	209	
*6.*	*Orai1 (NM_032790)*		*ATGGTGGCAATGGTGGAG*		*CTGATCATGAGCGCAAACAG*	*115*	
*7.*	*TRPC1 (NM_003304)*		*TTAGAGCTGGACTGGCCAAA*		*ATGCACATTGTGTTCTGCAA*	*95*	
*8.*	*STIM1 (NM_003156)*		*TGTGGAGCTGCCTCAGTATG*		*CTTCAGCACAGTCCCTGTCA*	*112*	
*8.*	*HPRT (NM_000194)*		*GGCGTCGTGATTAGTGATGAT*		*CGAGCAAGACGTTCAGTCCT*	*134*	

### Quantitative Real-time PCR

Quantitative real-time PCR of TRPC1, STIM1, Orai1 and HPRT mRNA transcripts was done using MESA GREEN qPCR MasterMix Plus for SYBR Assay (Eurogentec, France) on the Biorad CFX96 Real-Time PCR Detection System. The sequences of primers are indicated in Table 1. The HPRT gene was used as an endogenous control to normalize variations in RNA extractions, the degree of RNA degradation, and variability in RT efficiency. To quantify the results we used the comparative threshold cycle method ΔΔC(t).

### Reagents and Chemicals

All chemicals were purchased from Sigma (l’Isle d’Abeau, France) except fura-2/AM and thapsigargin (France Biochem, Meudon, France).

### Data Analysis and Statistics

Each experiment was repeated at least three times (n). The data were analyzed using PulseFit (HEKA Electronics, Germany) and Origin 7.0 (Microcal Software Inc., Northampton, MA, USA). Data are expressed as mean ± SEM. Statistical analysis was performed using ANOVA, and p<0.05 was considered as significant. Asterisks denote: * - p<0.05, and ** - p<0.01.

## Results

As it was currently well established that long-term androgen deprivation induces the NED of LNCaP cells [6], [8], [18], [34], we have used a 4-days androgen deprivation by means of charcoal stripped culture medium, in order to induce NED of LNCaP cells (NED-LNCaP). The functional results obtained on NED-LNCaP cells were compared with regular androgen-dependent LNCaP cells (CT-LNCaP), which served as a control.

### NED Downregulates Ca^2+^ Store-depletion and Subsequent Store-operated Ca^2+^ Entry

**Figure 1:**
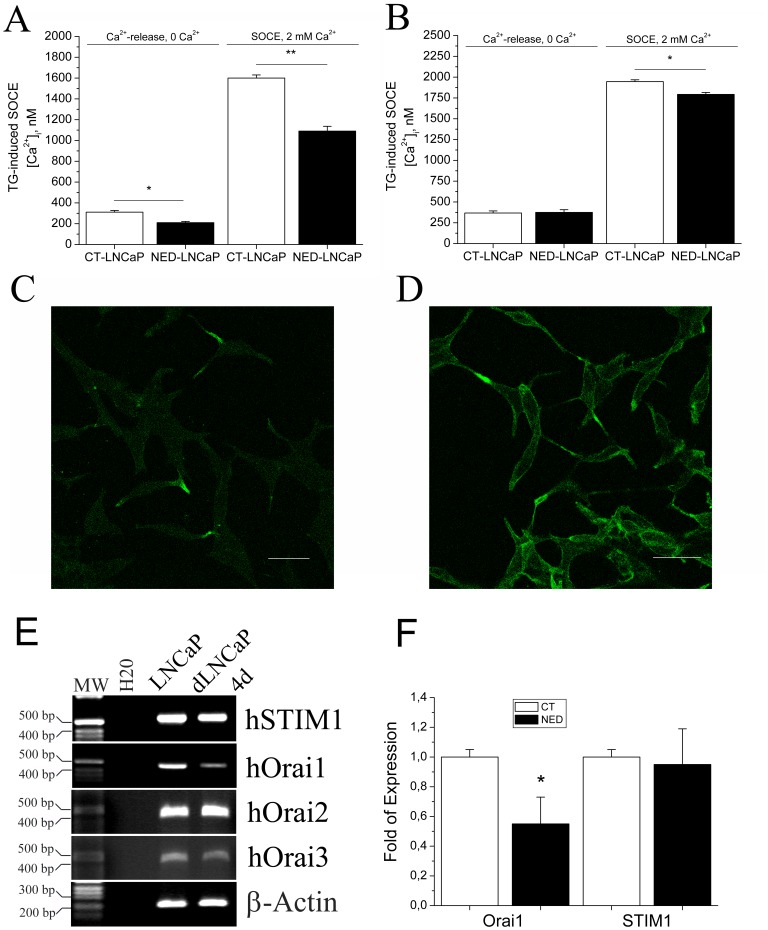
Regulation of Ca^2+^ homeostasis and F-actin polymerization in NED-LNCaP cells. After 4 (**A**) or one (**B**) days of culture in a charcoal-stripped culture medium used to induce NED, the capacitative Ca^2+^ entry quantified by Ca^2+^ imaging is induced by the application of 1 µM Tg in the presence of 2 mM extracellulaire CaCl_2._ Asterisks denote statistical significance * - p<0.05; ** - p<0.01, n = 3, N = 30–40 cells, in triplicates. The presence of F-actin fibres was observed in both CT-LNCaP (**C**) and NED-LNCaP (**D**) using phalloidin-FITC. Scale bar equals 10 µM. n = 3. **E**, Expression of Orai1, STIM1, Orai2, and Orai3 as compared to β-Actin in LNCaP cells and NED-LNCaP cells using semiquantitative PCR. n = 2. **F**, Quantitative real-time PCR for Orai1 and STIM1 in LNCaP (CT) versus LNCaP-NED (NED), n = 3, * - P<0.05.

Our first aim was to determine the effect of NED on endoplasmic reticulum (ER) Ca^2+^ stores filling status. The cells were first bathed in a Ca^2+^-free buffered solution and then exposed to a SERCA pumps inhibitor, thapsigargin (Tg at 1 µM). Ca^2+^ was released from ER Ca^2+^ stores into the cytosol by Tg, leading to a transient increase in intracellular Ca^2+^ concentration. Subsequently, 2 mM extracellular Ca^2+^ was added in the perfusion to permit capacitative calcium entry (SOCE) following store depletion. In these experimental conditions, the NED treatment did not significantly affect the basal Ca^2+^ level in the absence of extracellular Ca^2+^ (52±1.7 nM in CT-LNCaP *versus* 45±1.6 nM in NED-LNCaP, *n* = 240). In contrast, a 4-days NE differentiation treatment induced a ∼30% decrease in both store depletion and CCE (CT-LNCaP, 311±16 nM and 1600±30 nM, n = 180 *versus* NED-LNCaP, 210±11 nM and 1090±46 nM, n = 253, Fig. 1A). A one-day NED treatment only affected CCE (CT-LNCaP 1946±22, n = 152 *versus* NED-LNCaP 1793±23, n = 135, Fig. 1B). These results are in accord to our previous data showing that NED caused significant modifications in Ca^2+^ homeostasis, as a reduced filling of the ER Ca^2+^ store and substantial I_SOC_ down-regulation [18]. In the separate series of experiments we have checked the expression of SOCE key players as Orai1, Orai2, Orai3, and STIM1 using semiquantitative (Fig. 1E) and real-time quantitative PCR for Orai1 and STIM1 (Fig. 1F). Only Orai1 expression changed twice as we have previously published [20].

### NED Affects ER Ca^2+^ Stores Depletion through Actin Network Over-polymerization

**Figure 2:**
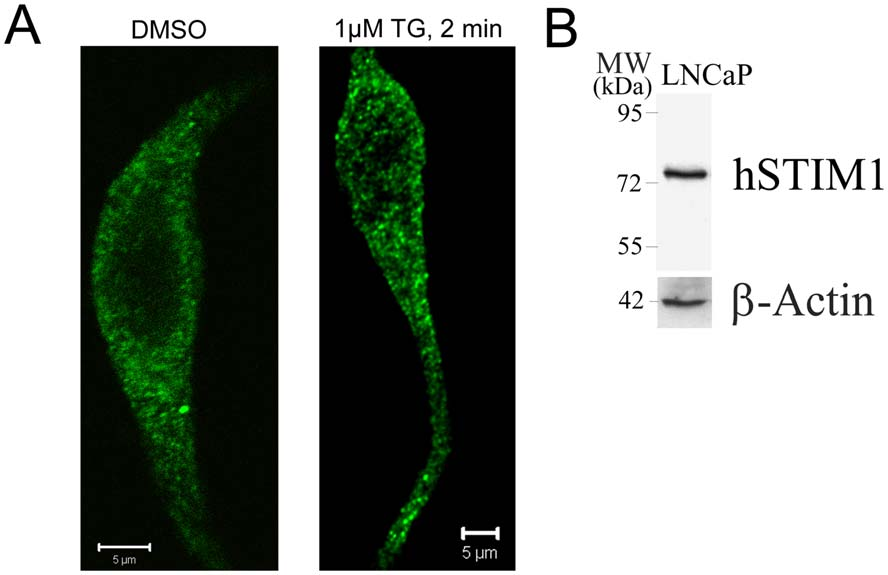
STIM1 forms punctae in NED-LNCaP cells upon induction of SOCE by TG. (**A**) NED-LNCaP cells stimulated either with DMSO (left panel) or treated with 1 µM TG for 2 min and stained with STIM1 antibody. Scale bar equals 5 µM. n = 3. (**B**) Western-blotting showing the expression of STIM1 protein in LNCaP cells using the anti-STIM1 antibody. n = 1.

During NED we and others have previously demonstrated that NED-LNCaP cells acquired dendritic-like extensions containing F-actin which is localized under the plasma membrane [7], [35], [36]. Our immunohistochemical staining using confocal microscopy showed over-polymerization of F-actin in NED-LNCaP cells (Fig. 1D) compared to CT-LNCaP cells (Fig. 1C). We have verified if in NED-LNCaP cells STIM1 is still able to form punctae upon ER Ca2+ store depletion. 1 µM TG was able to induce the formation of punctae upon 1 µM TG treatment during 2 minutes (Fig. 2). Thus, we have confirmed the formation of punctae by STIM1 in NED-LNCaP cells.

**Figure 3:**
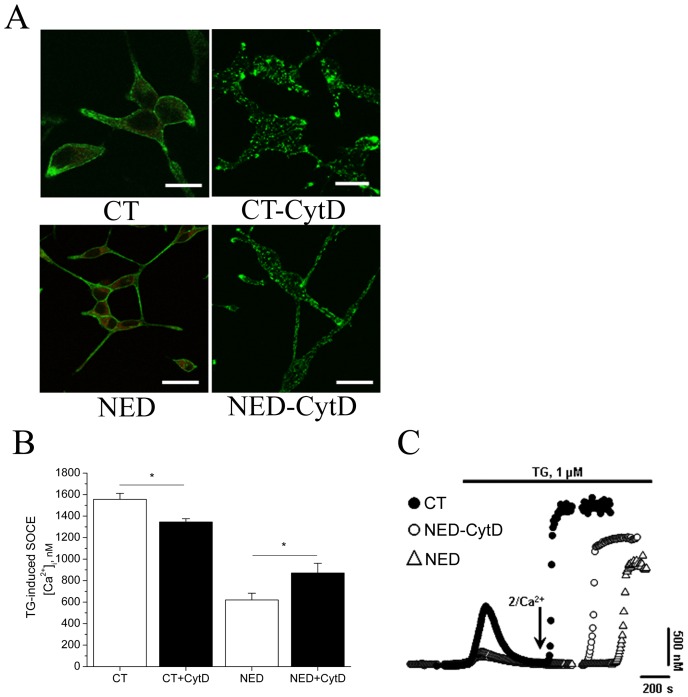
Cytochalasin D treatment disrupts the F-actin in both CT- and NED-LNCaP cells and partially restores the SOCE in differentiated LNCaP cells. (**A**) CT-LNCaP (top panel) and NED-LNCaP cells (bottom panel) were treated with 5 µM Cyt D for 10 min at 37°C and stained for F-actin using phalloidin-FITC. Scale bar equals 10 µM. n = 3. (**B**) Histograms representing Tg-induced SOCE quantification in CT-, CT-CytD-, NED- and NED-CytD-LNCaP cells. The SOCE was quantified by the application of 1 µM Tg in the presence of 2 mM extracellular Ca^2+^. n = 3, N = 30–40 cells, in triplicates, * p<0.05. (**C**) Intracellular Ca^2+^ concentration measured in fura-2 loaded cells showing that depolymerization of F-actin in NED-CytD-LNCaP treated cells partially restores the amplitude of the SOCE.

We have shown above that NED of LNCaP cells decreased the Tg-evoked SOCE (Fig. 1). Pharmacological treatments that destabilize the cortical actin network (CytD) prevent the activation of SOCE [37]. Using cytochalasin D (CytD, 3 µM, 10 min at 37°C) allows to interfere with the cytoskeleton and depolymerise the actin network [38] in both CT- and NED-LNCaP cells which leads to disappearance of long stress fibres in CT-LNCaP cells (Fig. 3A, top panel). Although stress fibres were broken, the neurite outgrowth of NED-LNCaP cells seemed to be always present, suggesting that NED may involve microtubules (Fig. 3A, bottom panel). The effects of CytD on SOCE of NED-LNCaP measured on-line as compared to NED-LNCaP only are shown in Fig. 3B. Depolymerization of F-actin by CytD (3 µM for 10 min at 37°C) decreased the Tg-induced SOCE in CT-LNCaP cells (1556±37 nM for CT, *n* = 33 versus 1345±31 nM for CT-CytD, *n* = 40) and partially restored the Tg-induced SOCE in NED-LNCaP (620±63 nM for NED, *n* = 33 versus 871±90 nM for NED-CytD, *n* = 42) (Fig. 3C). Depolymerization of actin does not have the same effect on SOCE according to the differentiation status of LNCaP cells.

**Figure 4:**
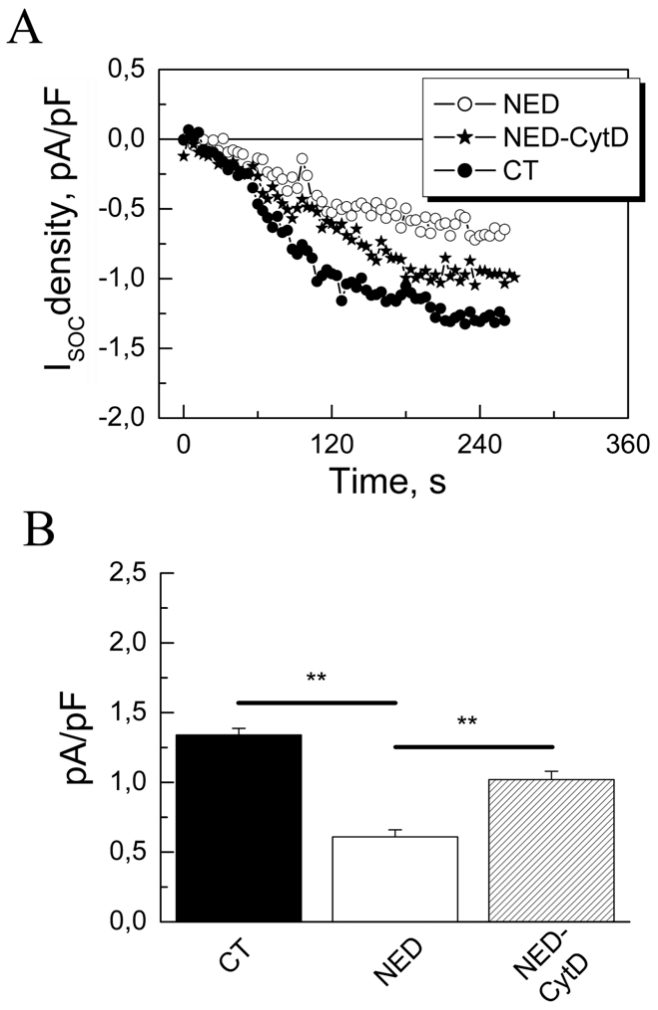
Cytochalasine D (CytD) partially restores the store-operated current induced by TG. (**A**) The whole-cell electrophysiological recordings of SOCE induced by 1 µM TG in LNCaP cells control (CT), NED (NED), and NED LNCaP cells treated with 3 µM CytD (NED-CytD). (**B**) Histograms showing the current density quantifications in the above conditions, n = 3, N = 23; in triplicates, ** - p<0.01.

In patch-clamp experiments we intended to see whether the observed effects of NED and actin depolymerisation on SOCE correlate with the store-operated membrane current. Using whole-cell electrophysiological recording and TG-evoked SOCE we have shown (Fig. 4) that NED largely suppresses store-operated membrane currents (0.61±0.05 for NED-LNCaP versus 1.34±0.047 pA/pF in control LNCaP, *n* = 23) and actin depolymerisation by CytD (3 µM, 10 min) only partially restores it (1.02±0.06 versus 0.061±0.05 pA/pF in NED-LNCaP, *n* = 23). These results suggest that the over-polymerization of the cortical F-actin observed during the NED may be a key event in the down-regulation of the Tg-induced SOCE in NED-LNCaP cells.

**Figure 5:**
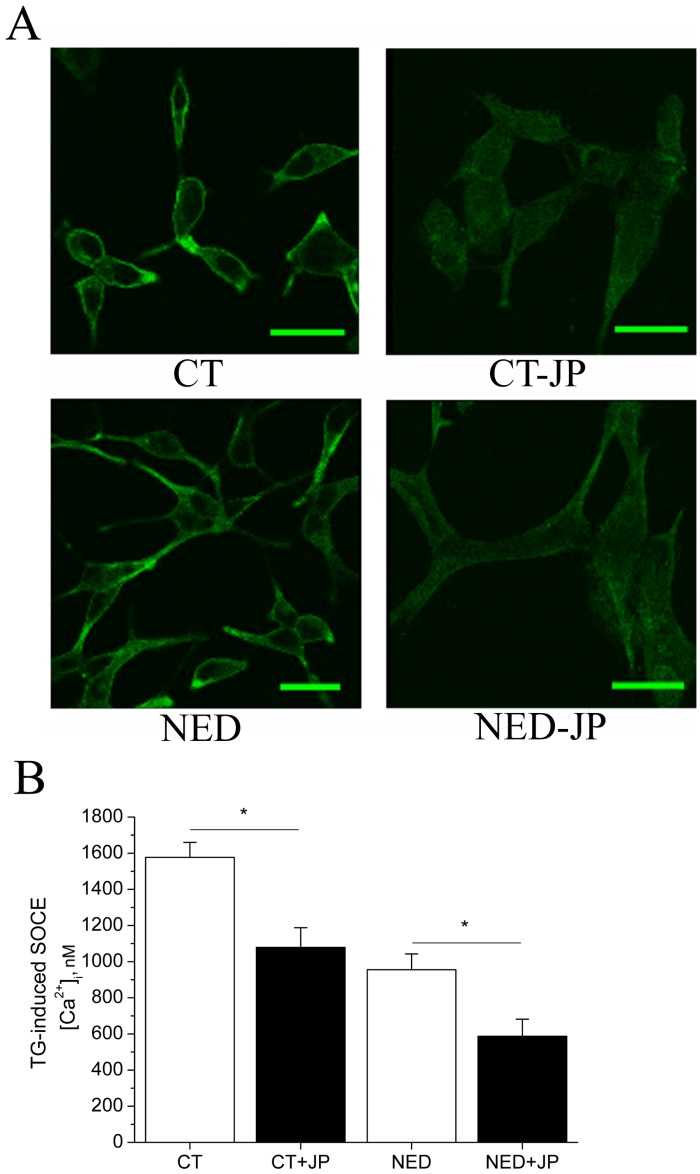
F-actin polymerization by jaspakinolide (JP) in both control and NED-LNCaP cells. (**A**) Representative images of immunofluorescence staining of F-actin with phalloidin-FITC in CT-, CT-JP (5 µM for 1 hour at 37°C), NED- and NED-JP-LNCaP cells. Scale bar equals 10 µM. n = 3. (**B**) Histograms representing Tg-induced SOCE quantification (Tg 1 µM, 2 mM extracellular Ca^2+^) in CT-, CT-JP-, NED- and NED-JP-LNCaP cells. n = 3, N = 30–40 cells, in triplicates. Asterisks denote statistical significance: * p<0.05.

To validate this hypothesis, we tested the effect of actin polymerization on Tg-evoked SOCE by using jasplakinolide (JP). The polymerizating effect of JP on actin is presented in CT- and NED-LNCaP cells (Fig. 5A). JP treatment (5 µM for 1 hour at 37°C) reduced SOCE amplitude in both CT- (1079±209 nM for CT-JP, *n* = 40 *versus* 1577±83 for CT, *n* = 44) and NED-LNCaP cells (955±88 nM for NED, *n* = 52 *versus* 587±95 nM for NED-JP, *n* = 52; Fig. 5B). The most interesting point to note is that the amplitude of SOCE in JP-CT-LNCaP cells, resulting from actin polymerization, is similar to the amplitude of SOCE in NED-LNCaP cells.

**Figure 6:**
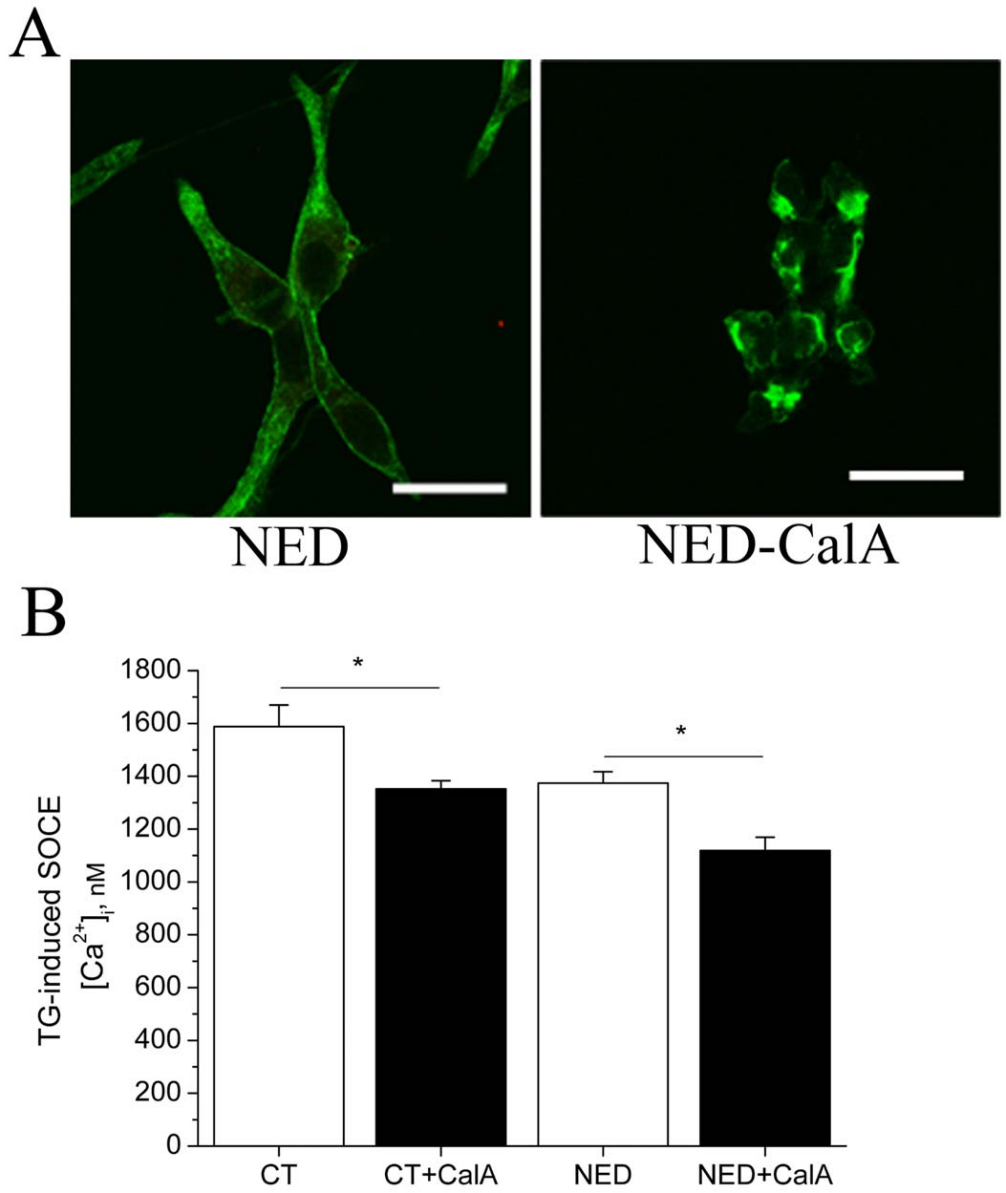
F-actin polymerization by calyculin A (CalA) in both control and NED-LNCaP cells. (**A**) Representative images of immunofluorescence staining of F-actin with phalloidin-FITC in NED- and NED-CalA (50 nM for 10 min)-LNCaP cells. n = 3. (**B**) Histograms representing Tg-induced SOCE quantification (Tg 1 µM, 2 mM extracellular Ca^2+^) in CT-, CT-CalA-, NED- and NED-CalA-LNCaP cells. n = 3, N = 30–40 cells, in triplicates. Asterisks denote statistical significance: * p<0.05.

Further, we used calyculin A (CalA) which specifically blocks PP1 and PP2A phosphatases, inducing cytoskeleton reorganization similar to that induced by JP [39]. In many cell types, actin either becomes condensed at the plasma membrane or condenses into actin bundles inside the cells [40], [41]. The polymerizating effect of CalA on actin is presented in NED-LNCaP cells (Fig. 6A). Identical action is observed in CT-LNCaP cells (data not shown). In both CT-LNCaP and NED-LNCaP cells, Tg-induced SOCE was decreased by a rapid treatment with CalA (50 nM for 10 min) (Fig. 6B) (1352±41 nM for CalA-CT, *n* = 74 *versus* 1588±52 nM for CT, *n* = 66) and in NED-LNCaP cells (1119±50 nM for CalA-NED, *n* = 56 *versus* 1374±43 nM for NED, *n* = 62). These observations matched those described with a JP treatment.

As it was previously mentioned, the most interesting finding is that JP-induced actin polymerization treatment mimics the down-regulatory effect of NED on SOCE in NED-LNCaP cells. This would indicate that the cytoskeleton reorganization may be one pathway used by NED to down-regulate SOC channels activity supporting SOCE in LNCaP cells.

**Figure 7:**
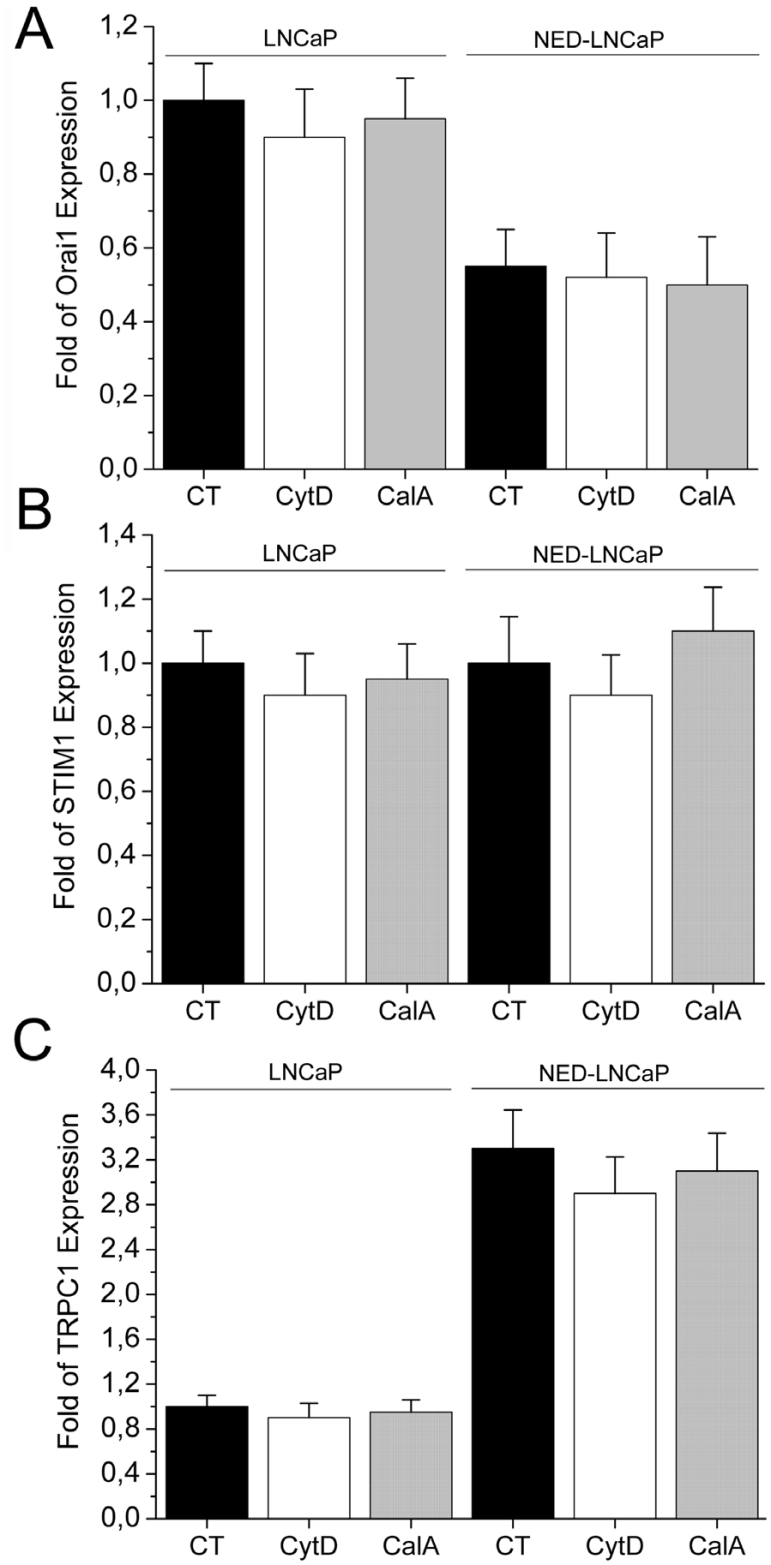
The expression of Orai1, STIM1 and TRPC1 does not change during CytD and CalA treatment in both LNCaP and NED-LNCaP. (**A**) Real-time quantitative PCR for the Orai1 transcripts in both LNCaP and NED-LNCaP cells treated with 50 nM CalA for 10 min or 5 µM Cyt D for 10 min. (**B**) Real-time quantitative PCR for the STIM1 transcripts in both LNCaP and NED-LNCaP cells treated with 50 nM CalA for 10 min or 5 µM Cyt D for 10 min. (**C**) Real-time quantitative PCR for the TRPC1 transcripts in both LNCaP and NED-LNCaP cells treated with 50 nM CalA for 10 min or 5 µM Cyt D for 10 min. There was no statistical significance observed in each condition (LNCaP and NED-LNCaP). n = 3, done in triplicates.

Finally, to prove that during our treatments no transcription *de novo* takes place we have performed a real-time quantitative PCR using the primers for Orai1, STIM1, and TRPC1 (Fig. 7). Cells (LNCaP and NED-LNCaP) were stimulated with either CytD, 3 µM, 10 min or CalA 50 nM for 10 min both at 37°C, RNA was extracted, reverse-transcribed and a real-time quantitative PCR was performed. As we can see from the Fig. 7, no significant changes in Orai1, STIM1, and TRPC1 expression were detected in either CytD or CalA treated cells. Thus the cytoskeleton reorganisation may take a significant role in modulation of membrane currents and SOCE in both control and NED prostate cancer cells.

## Discussion

In the present study we report two major findings characterizing the alternative pathway by which NED may contribute apoptosis resistance in PCa. Firstly, we describe that NED mediates SOCE down-regulation through cytosketon reorganization, especially F-actin over-polymerization. Secondly, we show that cytoskeleton reorganization is a pathway used by NED to down-regulate SOC channels activity without changing their expression.

### NED and Calcium Homeostasis Linked by Cytoskeleton

The activation of SOCE is a signalling process of great relevance as this mechanism is a key event in physiological processes such as cellular proliferation and apoptosis [42]. In pioneer works [18], [43], we showed that NED of LNCaP cells induced by long-term androgen-deprivation decreases the well-characterized store-operated Ca^2+^ current (I_SOC_). Here, we confirm by Ca^2+^ imaging measurements that NED in the same conditions down-regulated the SOCE amplitude. In addition, we also show that the Tg-induced SOCE decrease may result from a decrease in Ca^2+^ stores depletion in NED-LNCaP cells.

Despite these data, the activation mechanisms of SOCE are still unclear. However, three main hypothesis are proposed to explain SOC channels activity regulation: one related to a diffusible messenger termed CIF (Calcium Influx Factor) [44]), the second involves a conformational coupling between the plasma membrane [45] and the ER and the last one supporting an exocytosis-related translocation of SOC channels [24], [25], [27], [28], [29]. It is known that the two last mechanisms involve cytoskeleton rearrangement [46] as cytoskeleton has been shown to modulate SOCE [47], [48]. Indeed, the accumulation of F-actin into a cortical layer under plasma membrane after treatment with calyculin A and jasplakinolide prevents SOC channels activation in several models [49], [50]. In addition, cell morphology has been shown to play an essential role in agonist-induced ER Ca^2+^ stores depletion in endothelial cells [48]. We first studied the potential cytoskeleton involvement in ER Ca^2+^ store depletion as this process is the key event in SOCE induction [51], [52]. We and others have previously shown that NED causes the rearrangement of the cortical F-actin [18], [53]. Our data show that actin polymerization enhances, whereas actin depolymerization decreases Tg-induced Ca^2+^ stores depletion in undifferentiated LNCaP cells. Our results correlate with those obtained by Wang *et al*. (2002) on primary hippocampal cell cultures [54]. As both polymerizing (jasplakinolide and calyculin A) and depolymerizing agents (cytochalasine D) affect Tg-evoked Ca^2+^ stores release whatever the differentiation status of LNCaP cells, we suggest that the Ca^2+^ stores depletion down-regulation in NED-LNCaP cells does not results from the actin over-polymerization.

Concerning SOCE, as previously mentioned, cytoskeleton can regulate SOCE in many cellular types [49], [51], [55]. Although CytD induces filaments depolymerization, its predominant effect is the induction of filaments dissociation from the plasma membrane [49]. In this case, we show that depolymerization of cortical F-actin and its detachment from the plasma membrane refreshes the SOCE in NED-LNCaP cells. In contrast, the stimulation of the polymerization of the cortical F-actin by JP potentiates the SOCE amplitude decrease. Furthermore, the SOCE amplitude of JP-CT-LNCaP cells is identical to that of NED-LNCaP cells. This suggests that the polymerization of F-actin due to JP reproduces the NED effect on LNCaP cell’s cytoskeleton. The results obtained in control cells are in agreement with studies on vascular endothelial cells and human platelets [47], [50]. The conflicting effect produced by the CytD in control and NED cells can be explained by the percentage of F-actin contained in cells and suggests that in control cell, the cytoskeleton is required for channel function. This may also suggest the involvement of physical-coupling model in SOCE induction.

It has been suggested that Cal A activates a translocation of existing F-actin to the cell periphery, independent of polymerization and consistent with the phosphorylation events described above [49]. In our model, this translocation seems to be grouped around a pole of the cell. In these conditions, CalA failed to exactly reproduce the JP effect. Therefore, we suggest that, under NED, cortical F-actin is desorganized exclusively at the cell periphery. In summary, our data strongly suggest that the cytoskeleton reorganization may be another pathway used by NED to down-regulate SOC channels activity without changing their expression and consequently downregulate SOCE in LNCaP cells.

### Possible Mechanisms of SOCE-downregulation by NED

TRP (Transient Receptor Potential) protein family was shown to be involved in SOCE [46], [56], [57]. The mechanisms of TRP-channel regulation are still the subject of intense research as SOCE activation and down-regulation seems to be required in physiological processes such as immune response [27]. Several processes have been proposed to explain SOCE regulation: at the level of TRP proteins expression [58], activity regulation of TRP channels due to accessory proteins [59], [60] or by TRPC channels internalization/membrane insertion caused by cytoskeleton reorganization [24], [25], [26], [27], [28], [29]. TRP proteins are characterized by an ankyrin domain permitting the connection of proteins to the cytoskeleton. Consequently, modulations of the polymerization state of the cortical F-actin could modulate the activation of the TRP proteins. Indeed, Lockwitch et al. have shown that the status of the actin cytoskeleton affects the localization of the TRPC3-associated signalling complex. These authors suggest that stabilization of cortical actin induces internalization of TRPC3 and induces the loss of calcium influx [61]. Thus, TRP-channel internalization by the cytoskeleton reorganization may be proposed as a pathway used by NED to down-regulate SOC channels activity and consequently SOCE in prostate cancer cells.

### Implications for Androgen-independent Prostate Cancer

Apoptosis is essential in maintaining tissue homeostasis. The acquisition of a resistance to apoptosis plays a pivotal role in tumor genesis by disrupting the balance between cell proliferation and cell death. Androgen-independent prostate cancer is characterized by tumor enrichment in apoptosis resistant cell phenotypes as NED cells. Anti-apoptotic mechanisms involve basic changes in intracellular Ca^2+^ homeostasis as I_SOC_ down-regulation during the NED of prostate cancer epithelial cells [18]. In addition, we have previously identified TRPC1 and TRPC4 as proteins involved in Tg-evoked I_SOC_ in LNCaP cells [52], and demonstrated that NED LNCaP cells display a thapsigargin- (Tg) induced apoptosis resistance [18]. Our latest work showed Orai1 protein represents the major molecular component of endogenous store-operated Ca^2+^ entry in human prostate cancer cells, and constitutes the principal source of Ca^2+^ influx used by the cell to trigger apoptosis [20]. The functional coupling of STIM1 to Orai1, as well as Orai1 Ca^2+^-selectivity as a channel, is required for its pro-apoptotic effects we have also shown that the apoptosis resistance of androgen-independent PCa cells is associated with the downregulation of Orai1 expression as well as SOCE. Thought, in our recent work we clearly show that the alternative cytoskeleton-related mechanism may exist employing the downregulation of SOCE without changing the expression of neither Orai1 nor STIM1, nor TRPC1.

In conclusion, in the present study we show that the NED of LNCaP cells induced actin network over-polymerization resulting in SOCE down-regulation. Taken together, these results suggest the existence of an alternative regulation pathway for the control of SOCE which is most probably implicated in apoptosis-resistance of NED-LNCaP cells and consequently the apoptotic status of neuro-endocrine cells in advanced, androgen-independent prostate cancer.
